# The Potential Role of Human Papillomavirus Infection in Bell's Palsy: A Hypothesis-Generating Study Based on a Nationwide Cohort

**DOI:** 10.3389/fmed.2021.616873

**Published:** 2021-09-01

**Authors:** Kuan-Ying Li, Mei-Chia Chou, Renin Chang, Hei-Tung Yip, Yao-Min Hung, James Cheng-Chung Wei

**Affiliations:** ^1^Department of Neurology, Kaohsiung Medical University Hospital, Kaohsiung, Taiwan; ^2^Department of Neurology, School of Medicine, College of Medicine, Kaohsiung Medical University, Kaohsiung, Taiwan; ^3^Neuroscience Research Center, Kaohsiung Medical University, Kaohsiung, Taiwan; ^4^Shu-Zen Junior College of Medicine and Management, Kaohsiung, Taiwan; ^5^Department of Recreation and Sports Management, Tajen University, Pingtung, Taiwan; ^6^Department of Physical Medicine and Rehabilitation, Kaohsiung Veterans General Hospital, Pingtung, Taiwan; ^7^Graduate Institute of Bioresources, National Pingtung University of Science and Technology, Pingtung, Taiwan; ^8^Department of Emergency Medicine, Kaohsiung Veterans General Hospital, Kaohsiung, Taiwan; ^9^Institute of Medicine, Chung Shan Medical University, Taichung, Taiwan; ^10^Management Office for Health Data, China Medical University Hospital, Taichung, Taiwan; ^11^College of Medicine, China Medical University, Taichung, Taiwan; ^12^Institute of Public Health (Biostatistics), National Yangming University, Taipei, Taiwan; ^13^Department of Internal Medicine, Kaohsiung Municipal United Hospital, Kaohsiung, Taiwan; ^14^College of Health and Nursing, Meiho University, Pingung, Taiwan; ^15^National Yang-Ming University, School of Medicine, Taipei, Taiwan; ^16^Division of Allergy, Immunology and Rheumatology, Chung Shan Medical University Hospital, Taichung, Taiwan; ^17^Graduate Institute of Integrated Medicine, China Medical University, Taichung, Taiwan

**Keywords:** Bell's palsy, human papillomavirus, cohort study, infection, HPV

## Abstract

**Objective:** Our purpose was to investigate whether people with a previous human papillomavirus (HPV) infection were associated with an increased risk of Bell's palsy (BP).

**Methods:** By using Taiwan population-based data, patients aged > 18 years with HPV infection (*n* = 22,260) from 2000 to 2012 were enrolled and compared with control subjects who had never been diagnosed with an HPV infection at a 1:4 ratio matched by sex, age, index date, and co-morbidities (*n* = 89,040). The index date was the first date of HPV diagnosis. All the patients were tracked until the occurrence of BP. Cox proportional hazards regression was applied to estimate the hazard ratios (HRs) for the development of BP in both groups.

**Results:** The HPV group had 1.25 [95% confidence interval (CI) = 1.03–1.51] times higher risk of BP compared with the non-HPV group after adjusting for sex, age, and co-morbidities. The association of HPV and BP was significant in the sensitivity analyses. In the subgroup analysis, the impact of HPV infection on the risk of BP was more pronounced in the elderly > 50 years [adjusted hazard ratio (aHR) =1.86; 95% CI = 1.37–2.52], hypertension (aHR = 1.65; 95% CI = 1.17–2.31), and chronic obstructive pulmonary disease (aHR = 2.14, 95% CI 1.333.43) subgroups.

**Conclusions:** Patients with HPV infection have a higher risk of subsequent BP compared with non-HPV patients. More rigorous studies are needed to confirm if and how specific HPV genotypes are associated with BP and the possible role of vaccines in disease prevention.

## Introduction

Bell's palsy (BP), defined as acute peripheral facial nerve palsy of unknown cause, is the most common acute mono-neuropathy (1). This disease affects 11–40 people per 100,000 annually (2). Typically, BP results in a partial or complete inability to move the affected side of the facial muscles. There is no race, geographic, or gender preference (3). The risk factors include diabetes, hypertension, and pregnancy (3, 4). Although the facial paresis or paralysis usually resolves within several months, permanent symptoms may occur and affect the quality of life of the patient (5). Indeed facial paralysis was significantly associated with increased depression, anxiety, and a worse quality of life (6). The cause of BP remains unknown, and many events, such as ischemia, viral infection, acute cold exposure, and anatomic causes, have been proposed as causes of BP (7). Investigators proposed that immune alterations related to a previous viral infection have an important role in the etiopathogenesis of BP (8). The reasons for this include alterations in lymphocyte subsets in the peripheral blood during the acute stage of the disease (9, 10). Additionally, lymphocytes from patients with BP were stimulated in the presence of neuritogenic basic protein from the peripheral nerve myelin sheath (11). A similar pattern of response was also demonstrated in patients with Guillain–Barre syndrome (12, 13), an autoimmune demyelination neuritis triggered by a preceding infection (14). However, serological evidence and histopathology of facial nerves support the infectious nature of BP. Herpes simplex viruses 1 and 2 and varicella-zoster viruses are the most common viruses associated with BP (15). Other infections, such as Epstein–Barr virus, cytomegalovirus, and human immunodeficiency virus, have also been reported to be associated with BP (15–17). Human papillomaviruses (HPV) are small double-stranded DNA viruses known as risk factors for multiple cancers (18–20) and are also associated with autoimmune diseases, such as systemic lupus erythematosus (SLE) and rheumatoid arthritis (RA) (21–23). The shared pathogenesis between these autoimmune diseases and BP is based on a viral infection and a subsequent autoimmune reaction (24, 25). These findings suggest a possible association between HPV infection and BP. However, there is no epidemiologic evidence to support this hypothesis. Thus, we conducted this original nationwide population-based cohort study to assess the incidence rate and risk of BP in people with a history of HPV infection when compared with those without HPV infection.

## Methods

### Data Source

The data in this study were obtained from Taiwan's National Health Insurance Research Database (NHIRD), which contains registration files and original claim data, such as diagnoses coding, out-patient visits, hospitalization records, and medication and personal information, for almost 99% of the population of Taiwan. Moreover, ~93% of healthcare facilities in Taiwan have signed contracts with the Bureau of National Health Insurance. To ensure the accuracy of these data, all claims data were validated by the Bureau of National Health Insurance. The data used in the study were obtained from the Longitudinal Health Insurance Database (LHID), a subset of NHIRD. The LHID contains original claims data of 1,000,000 people randomly sampled from the 1996 to 2013 registries for beneficiaries of the NHIRD, and the dataset has been used for medical and epidemiological research (26). There were no statistically significant differences in the distribution of age, sex, or healthcare costs between the two databases. With a strict definition of the study group selection, the positive predictive value of the NHIRD claim data can be up to 84.6%. The authority replaced the original identification numbers with surrogate numbers before the data were released to protect the privacy of the people. The Institutional Review Board of the China Medical University in Taiwan approved this study [CMUH140-REC2-115 (CR-4)].

### Data Availability Statement

Data are available from the NHIRD published by Taiwan National Health Insurance (NHI) Bureau. Due to legal restrictions imposed by the government of Taiwan in relation to the “Personal Information Protection Act,” data cannot be made publicly available. Requests for data can be sent as a formal proposal to the NHIRD (http://nhird.nhri.org.tw).

### Study Population

**Figure 1:**
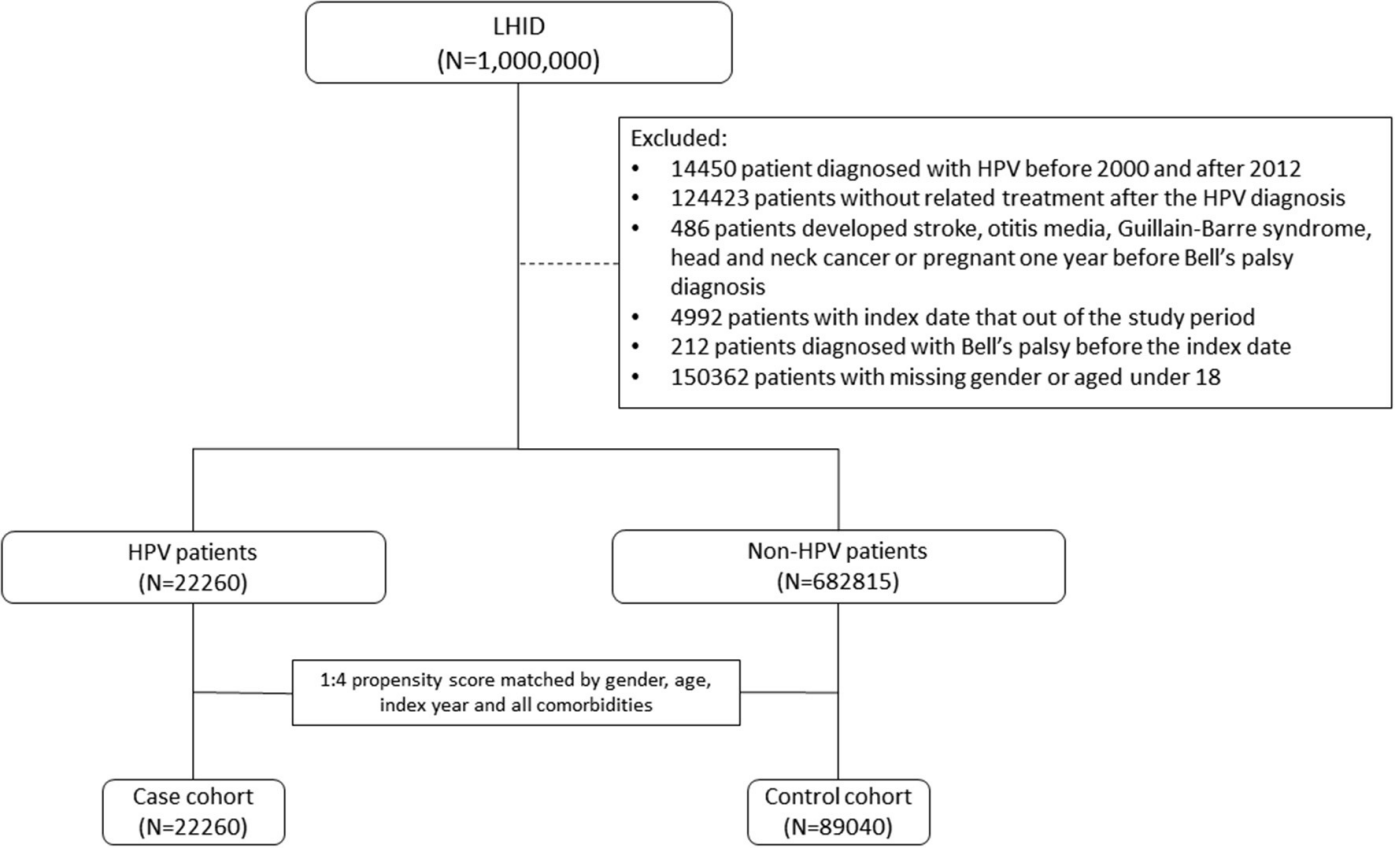
Flow chart of the cohort assembly in the current study.

To ensure the accuracy of patient information during the study period, patients with missing demographic data and those who died before 2003 were excluded from this analysis. HPV infection was defined according to the International Classification of Diseases, Ninth Revision, Clinical Modification (ICD-9-CM) codes. We identified patients who had been diagnosed with HPV infection (ICD-9-CM codes 079.4, 078.1, 078.10–078.12, 078.19, 795.05, 795.09, 795.15, 795.19, 796.75, and 796.79) between 2000 and 2012 as the HPV group. The index date was defined as the date of HPV infection diagnosis. For each patient in the study group, the corresponding controls were selected at a ratio of 1:4 based on the nearest propensity score. The propensity scores were calculated using the probability of HPV assignment by using a logistic regression model and included the following baseline variables: year of index date; age; sex; comorbidities of hypertension, diabetes, hyperlipidemia, chronic kidney disease, and chronic obstructive pulmonary disease (COPD); alcohol-related illness; herpes simplex virus type 1; herpes zoster; systemic lupus erythematosus; rheumatoid arthritis; Sjögren's syndrome; and multiple sclerosis. Figure 1 shows the exclusion criteria and process of assembling the HPV group and the non-HPV group step by step. To ameliorate potential confounders in the analysis, patients with well-known risk factors contributing to BP, such as stroke, otitis media, Guillain–Barre syndrome, and head and neck cancer, or pregnancy 1 year before BP were excluded (Figure 1). The Supplementary Figure 1 shows an overview of the study. Data from 1996 to 2000 in the dataset were used as a wash-out period to ensure that the patients in both groups did not have a record of BP or HPV infection before study entry. To strengthen the validity of coding for HPV-associated diseases, patients with an HPV infection who did not receive any HPV-related treatment procedures in the 3 months after the index date were excluded. Such treatment procedure codes included “electrocauterization for condyloma (50005),” “condyloma, excision, and electrocauterization (55008),” “CO_2_ laser operation (62020),” “chemosurgery, condyloma (50015),” “electro-cauterization, simple (51005),” “electro-cauterization, complicated (51006),” “liquid nitrogen cryosurgery (51017),” “cryotherapy, simple, including CO_2_ freezing and liquid nitrogen (51021C),” and “cryotherapy, complicated, including CO_2_ freezing and liquid nitrogen (51022).” All participants were followed until the presence of BP, dropping out from the insurance program, death, or the end of the study (December 31, 2013). Finally, 22,260 subjects were included in the HPV group and 89,040 subjects served as the control group.

### Covariates and Outcome

The co-morbidities analyzed in this study were hypertension (ICD-9-CM codes 401–405), diabetes mellitus (ICD-9-CM code 250^**^), hyperlipidemia (ICD-9-CM code 272), chronic obstructive pulmonary disease (ICD-9-CM codes 490–492 and 493–496), and herpes zoster (ICD-9-CM code 053^**^). All subjects in both groups were tracked from the index date to the first BP event. The end-point of this study was the occurrence of BP (ICD-9-CM code 351.0) (27). To improve the reliability of ICD coding, a BP event was defined as a patient having a record of BP diagnosis in three or more ambulatory visits or at least one admission within a year. The co-morbidities were obtained in the LHID 2 years before the index date of HPV diagnosis.

### Statistical Analysis

First, we applied the chi-square test to compare the distribution of age, sex, and baseline co-morbidities between the two groups, with or without previous HPV infection. We compared the mean age using Student's *t*-test. The incidence density of BP per 1,000 person-years was calculated for both groups.

Second, we utilized multivariable Cox proportional hazard regression models to estimate the crude hazard ratios and adjusted HRs (aHRs) of BP and related 95% confidence intervals (CIs) of the HPV group compared to the non-HPV group adjusting for sex, age, and the aforementioned co-morbidities. We calculated the receiver operating characteristic curve for the main regression model and its area under the curve to assess the fit of the model. Two sensitivity analyses were conducted, including data on concomitant medication with anti-inflammatory effects and medical conditions that might be associated with immune system functions. In the first sensitivity analysis, potential anti-inflammatory medications were included in the covariates (Supplementary Tables 1, 2); in the second sensitivity analysis, we changed different coexisting medical co-morbidities as covariates (Supplementary Tables 3, 4).

Third, the Kaplan–Meier method was used to plot the cumulative incidence curves of BP for both groups. Differences between the two groups were evaluated by log-rank test.

Fourth, to reveal the association of sex, age, and follow-up time on the incidence rate of BP, we applied multivariable Cox regression models adjusted for sex, age, and co-morbidities. The HRs adjusted for covariates were calculated for males and females; age (years) <30, 31–40, 41–50, and above 50; and follow-up time <1 year, 1–5 years, and >5 years. All data analyses were performed with SAS® (version 9.4; SAS Institute, Inc., Cary, NC, USA). The statistical significance level was set at *P*-value < 0.05 by two-tailed test.

## Results

### Cohort Characteristics

The study consisted of 89,040 non-HPV subjects and 22,260 HPV subjects. The baseline characteristics of the two groups are listed in Table 1. In both groups, there was a slight female predominance (52%), and subjects aged below 30 years old (35%) comprised the largest age group.

**Table 1:** Baseline patient characteristics.

	**Non-HPV**		**HPV**		
	***N*** **=** **89,040**		***N*** **=** **22,260**		
**Variables**	***n***	**%**	***n***	**%**	**SMD**
Sex					<0.001
Female	46,648	52%	11,662	52%	
Male	42,392	48%	10,598	48%	
**Age, year**
<30	30,760	35%	7,690	35%	<0.001
30–39	1,8076	20%	4,519	20%	<0.001
40–49	16,492	19%	4,123	19%	<0.001
50–59	11,488	13%	2,872	13%	<0.001
60–69	6,020	7%	1,505	7%	<0.001
70–79	4,444	5%	1,111	5%	<0.001
≥80	1,760	2%	440	2%	<0.001
Mean (SD)	40.4	(16.6)	40.4	(16.6)	0.003
**Comorbidities**
Hypertension	14,804	17%	4,029	18%	0.04
Diabetes	7,380	8%	1,991	9%	0.02
Hyperlipidemia	12,856	14%	4,048	18%	0.10
CKD	934	1.0%	325	1.5%	0.04
COPD	7,317	8.2%	2,291	10%	0.07
Alcohol-related illness	1,825	2.0%	484	2.2%	0.01
HSV1	4,706	5.3%	1,961	8.8%	0.14
Herpes zoster	2,469	2.8%	905	4.1%	0.07
SLE	241	0.27%	97	0.44%	0.03
Rheumatoid arthritis	1,456	1.6%	455	2.0%	0.03
Sjögren's syndrome	666	0.7%	302	1.4%	0.06
multiple sclerosis	27	0.03%	3	0.01%	0.01

*CKD, chronic kidney disease; COPD, chronic obstruction pulmonary disease; HSV1, genital herpes simplex virus; SLE, systemic lupus erythematosus*.

### Risk of BP

**Figure 2:**
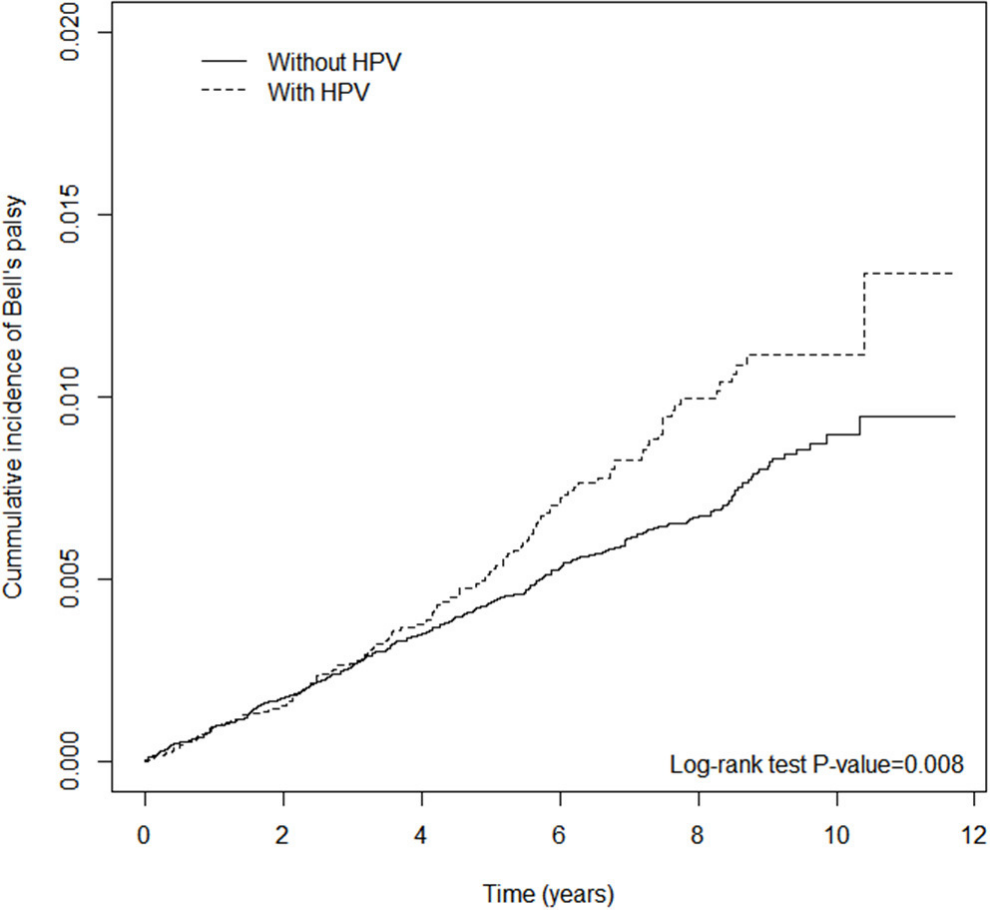
Cumulative incidence of Bell's palsy for the human papillomavirus (HPV) and the non-HPV.

Table 2 shows the incidence rate and HRs for BP. There were 569 cases of newly diagnosed BP. The crude HR of HPV was 1.29 (95% CI = 1.07–1.57); the aHR after adjusting for demographic variables, including sex, age, and co-morbidities, was 1.25 (95% CI = 1.03–1.51). The risk of BP was higher in subjects above the age of 30 years, especially those in the age groups 30–39 and 40–49 years, with 1.36 (95% CI = 1.06–1.73) and 1.42 (95% CI = 1.10–1.81) times higher risk compared with individuals under 30 years old. The association between HPV infection and BP remained significant after considering coexisting medical conditions and medications (Supplementary Tables 2, 4). There were no statistically significant differences in risk between males and females. In Figure 2, the cumulative incidence curves plotted by the Kaplan–Meier method shows that the HPV group had a higher cumulative incidence of BP than that of the non-HPV group (log-rank test *P-*value < 0.008).

**Table 2:** Bell's palsy incidence rate and risk factors.

	**Bell's palsy**						
**Variables**	***n***	**PY**	**IR**	**cHR**	**(95% CI)**	**aHR^†^**	**(95% CI)**
**HPV**
No	429	488,742	0.88	1.00	–	1.00	–
Yes	140	123,173	1.14	1.29	(1.07, 1.57)^**^	1.25	(1.03, 1.51)^*^
**Sex**
Female	301	322,858	0.93	1.00	–		
Male	268	289,057	0.93	0.99	(0.84, 1.17)		
**Age, year**
<30	145	222,648	0.65	1.00	–	1.00	–
30–39	116	126,637	0.92	1.41	(1.10, 1.80)^**^	1.36	(1.06, 1.73)^*^
40–49	119	113,474	1.05	1.62	(1.27, 2.06)^***^	1.42	(1.10, 1.81)^**^
≥50	189	149,157	1.27	1.96	(1.58, 2.44)^***^	1.26	(0.96, 1.65)
**Comorbidities**
Hypertension							
No	417	519,255	0.80	1.00	–	1.00	–
Yes	152	92,660	1.64	2.06	(1.71, 2.48)^***^	1.51	(1.18, 1.93)^**^
Diabetes							
No	489	566,474	0.86	1.00	–	1.00	–
Yes	80	45,441	1.76	2.05	(1.62, 2.6)^***^	1.29	(0.98, 1.71)
Hyperlipidemia							
No	436	527,717	0.83	1.00	–	1.00	–
Yes	133	84,198	1.58	1.92	(1.58, 2.34)^***^	1.26	(0.99, 1.61)
CKD							
No	563	606,820	0.93	1.00	–		
Yes	6	5,095	1.18	1.28	(0.57, 2.87)		
COPD							
No	498	563,832	0.88	1.00	–	1.00	–
Yes	71	48,083	1.48	1.68	(1.31, 2.15)^***^	1.19	(0.91, 1.55)
Alcohol-related illness							
No	555	601,547	0.92	1.00	–		
Yes	14	10,368	1.35	1.48	(0.87, 2.52)		
HSV1							
No	547	580,042	0.94	1.00	–		
Yes	22	31,873	0.69	0.74	(0.48, 1.13)		
Herpes zoster							
No	543	596,548	0.91	1.00	–	1.00	–
Yes	26	15,367	1.69	1.88	(1.27, 2.78)^**^	1.45	(0.97, 2.16)
SLE							
No	567	610,183	0.93	1.00	–		
Yes	2	1,732	1.15	1.25	(0.31, 5.01)		
Rheumatoid arthritis							
No	558	602,502	0.93	1.00	–		
Yes	11	9,413	1.17	1.27	(0.7, 2.31)		
Sjögren's syndrome							
No	564	607,605	0.93	1.00	–		
Yes	5	4,310	1.16	1.27	(0.52, 3.05)		
Multiple sclerosis							
No	569	611,785	0.93	1.00	–		
Yes	0	130	0.00	0.00	(0, Inf)		

* *p-value < 0.05;*

** *p-value < 0.01;*

*** *p-value < 0.001*.

*PY, person-years; IR, incidence rate (per 1,000 person-years); cHR, crude hazard ratio; aHR, adjusted hazard ratio; CKD, chronic kidney disease; COPD, chronic obstruction pulmonary disease; HSV1, genital herpes simplex virus; SLE, systemic lupus erythematosus*.

† *Adjusted by age, hypertension, diabetes, hyperlipidemia, COPD and herpes zoster*.

### Subgroup Analysis

Table 3 shows the incidence rate and aHRs for BP among different subgroups stratified by sex, age, and follow-up time. The interaction between sex and HPV infection on the risk of BP was not significant (*P*-value for interaction = 0.55). The proportion of individuals with BP was highest in patients aged > 50 years in both groups. When we compared the risk of BP in both groups according to age, those aged > 50 years had the highest statistically significant risk of developing BP (aHR = 1.86; 95% CI = 1.37–2.52). The interaction between age and HPV infection on the risk of BP was significant (*P*-value for interaction = 0.01). In the comorbidity subgroup analysis, a higher relative risk for BP in the HPV group compared with the control group was observed for the subgroup with hypertension (aHR 1.65; 95% CI = 1.17–2.31; *P*-value for interaction = 0.04) and COPD (aHR 2.14; 95% CI = 1.33–3.43; *P*-value for interaction = 0.01). When stratified by follow-up time (Table 4), the HPV group had a significantly higher aHR for BP > 5 year (aHR = 1.61; 95% CI = 1.15–2.26). No statistically significant risks were found in follow-up time <1 year and 1–5 years.

**Table 3:** Subgroup analysis.

	**Non-HPV**			**HPV**							
**Variables**	**Event**	**PY**	**IR**	**Event**	**PY**	**IR**	**cHR**	**(95% CI)**	**aHR^†^**	**(95% CI)**	***P*-value for interaction**
Sex											0.55
Female	230	257,929	0.89	71	64,929	1.09	1.23	(0.94, 1.6)	1.19	(0.91, 1.56)	
Male	199	230,813	0.86	69	58,244	1.18	1.37	(1.04, 1.81)^*^	1.33	(1.01, 1.75)^*^	
Age, year											0.01
<30	118	178,091	0.66	27	44,557	0.61	0.91	(0.6, 1.39)	0.91	(0.60, 1.39)	
30–39	92	101,190	0.91	24	25,446	0.94	1.04	(0.66, 1.63)	1.00	(0.64, 1.57)	
40–49	93	90,619	1.03	26	22,855	1.14	1.11	(0.72, 1.71)	1.07	(0.69, 1.66)	
≥50	126	118,842	1.06	63	30,315	2.08	1.95	(1.44, 2.65)^***^	1.86	(1.37, 2.52)^***^	
**Co-morbidities**
Hypertension											0.04
No	327	416,603	0.78	90	102,652	0.88	1.12	(0.88, 1.41)	1.10	(0.87, 1.39)	
Yes	102	72,139	1.41	50	20,521	2.44	1.71	(1.22, 2.4)^**^	1.65	(1.17, 2.31)^**^	
Diabetes											0.43
No	373	453,225	0.82	116	113,249	1.02	1.24	(1.01, 1.53)^*^	1.21	(0.98, 1.49)	
Yes	56	35,518	1.58	24	9,924	2.42	1.53	(0.95, 2.47)	1.50	(0.93, 2.42)	
Hyperlipidemia											0.47
No	338	425,311	0.79	98	102,406	0.96	1.20	(0.96, 1.51)	1.20	(0.96, 1.51)	
Yes	91	63,431	1.43	42	20,767	2.02	1.41	(0.97, 2.03)	1.38	(0.95, 1.99)	
CKD											0.36
No	426	485,024	0.88	137	121,795	1.12	1.28	(1.06, 1.55)^*^	1.24	(1.02, 1.5)^*^	
Yes	3	3,718	0.81	3	1377	2.18	2.68	(0.54, 13.29)	2.58	(0.51, 13.17)	
COPD											0.01
No	388	452,620	0.86	110	111,212	0.99	1.15	(0.93, 1.43)	1.13	(0.91, 1.39)	
Yes	41	36,123	1.14	30	11,961	2.51	2.21	(1.38, 3.54)^***^	2.14	(1.33, 3.43)^**^	
Alcohol-related illness											0.33
No	417	480,569	0.87	138	120,978	1.14	1.31	(1.08, 1.59)^**^	1.27	(1.04, 1.54)^*^	
Yes	12	8,173	1.47	2	2,195	0.91	0.62	(0.14, 2.77)	0.62	(0.14, 2.79)	
HSV1											0.62
No	414	466,656	0.89	133	113,386	1.17	1.32	(1.09, 1.61)^**^	1.28	(1.05, 1.56)^*^	
Yes	15	22,086	0.68	7	9,787	0.72	1.07	(0.44, 2.62)	1.01	(0.41, 2.49)	
Herpes zoster											0.62
No	413	477,767	0.86	130	118,781	1.09	1.27	(1.04, 1.54)^*^	1.23	(1.01, 1.5)^*^	
Yes	16	10,975	1.46	10	4,392	2.28	1.56	(0.71, 3.44)	1.52	(0.69, 3.37)	
SLE											0.94
No	427	487,525	0.88	140	122,658	1.14	1.30	(1.08, 1.58)^**^	1.26	(1.04, 1.52)^*^	
Yes	2	1,218	1.64	0	514	0.00	0.00	(0, Inf)	0.00	(0, Inf)	
Rheumatoid arthritis											0.89
No	421	481,604	0.87	137	120,898	1.13	1.30	(1.07, 1.57)^**^	1.25	(1.03, 1.52)^*^	
Yes	8	7,138	1.12	3	2,275	1.32	1.20	(0.32, 4.51)	1.06	(0.28, 4.01)	
Sjögren's syndrome											0.92
No	426	485,807	0.88	138	121,798	1.13	1.29	(1.07, 1.57)^**^	1.25	(1.03, 1.51)^*^	
Yes	3	2,935	1.02	2	1,375	1.45	1.42	(0.24, 8.47)	1.25	(0.21, 7.62)	
Multiple sclerosis											1.00
No	429	488,628	0.88	140	123,157	1.14	1.29	(1.07, 1.57)^**^	1.25	(1.03, 1.51)^*^	
Yes	0	114	0.00	0	16	0.00					

* *p-value < 0.05;*

** *p-value < 0.01;*

*** *p-value < 0.001*.

*PY, person-years; IR, incidence rate (per 1,000 person-years); cHR, crude hazard ratio; aHR, adjusted hazard ratio; CKD, chronic kidney disease; COPD, chronic obstruction pulmonary disease; HSV1, genital herpes simplex virus; SLE, systemic lupus erythematosus*.

† *Adjusted by age, hypertension, diabetes, hyperlipidemia, COPD and herpes zoster*.

**Table 4:** Bell's palsy in different follow-up period.

	**Non-HPV**			**HPV**							
	**Event**	**PY**	**IR**	**Event**	**PY**	**IR**	**cHR**	**(95% CI)**	**aHR^†^**	**(95% CI)**	***P*-value for interaction**
Follow-up time, year											0.28
<1	85	87,769	0.98	21	21,994	0.95	1.00	(0.62, 1.61)	0.97	(0.60, 1.57)	
1–5	231	360,538	0.64	71	90,833	0.78	1.22	(0.93, 1.59)	1.17	(0.90, 1.53)	
>5	113	369,308	0.31	48	93,391	0.51	1.68	(1.2, 2.36)^**^	1.61	(1.15, 2.26)^**^	

** *p-value < 0.01*.

*PY, person-years; IR, incidence rate (per 1,000 person-years); cHR, crude hazard ratio; aHR, adjusted hazard ratio*.

† *Adjusted by age, hypertension, diabetes, hyperlipidemia, COPD and herpes zoster*.

### Receiver Operating Characteristic Curve

Finally, we calculated the receiver operating characteristic curve for the main regression model in years 1, 5, and 10 and the area under the curve to assess the fit of the model. The area under the curve increased with the increase in time, indicating that the model could predict the risk of BP (Supplementary Figure 2).

## Discussion

In this population study, people with HPV infection had a significantly increased risk of BP compared with the sex-, age-, and index date-matched non-HPV group. After adjusting for sex, age, and co-morbidities, the HPV group had a 25% increased risk of developing BP compared with the non-HPV group. The consistency of a positive association from different sensitivity analyses validated our findings. In our stratified analysis, we found a prominent interaction effect between age and HPV infection on the risk of new-onset BP. Among participants aged 50 years and older, having an HPV infection associated with a higher risk of developing BP than those without an HPV infection. We also observed significant interactions between HPV infection and some comorbidities (hypertension and COPD). The simultaneous presence of HPV infection and hypertension or COPD revealed a higher aHR than HPV infection alone.

Our study showed consistent findings in reporting the epidemiology of BP with previous studies. In Table 2, the incidence rate was similar in women and men, which was compatible with a previous study in the UK (28). Our study showed that patients aged above 30 years were more susceptible to BP. This result is in agreement with a previous study that showed that BP was more common in mid- and late-life, with the highest incidence in the fifth decade of life (29). We reported that hypertension increased the risk of developing BP by 50%, which is in accord with a previous case–control study that enrolled 201 patients with BP aged over 40 years or older and reported a 4.5-fold increase in the odds of BP being associated with hypertension (30).

The underlying mechanism by which an HPV infection increases the risk of developing BP remains unclear. However, an HPV infection was associated with autoimmune diseases, such as SLE, RA, and psoriasis (31, 32). A previous study showed that an HPV infection triggered SLE *via* molecular mimicry (22) because the HPV protein has a high and widespread similarity to several human proteins (21, 33). Similarly, molecular mimicry plays an important role in the immunopathogenic mechanisms of Guillain–Barré syndrome and related diseases (14). An antecedent infection evokes an immune response, which, in turn, cross-reacts with peripheral nerve components due to the sharing of cross-reactive epitopes. The most common identified triggering agents are *Campylobacter jejuni, Mycoplasma pneumoniae*, Cytomegalovirus, and Epstein–Barr virus. All of these pathogens have carbohydrate sequences in common with peripheral nerve tissues (34). Therefore, molecular mimicry might explain how an HPV infection increases the risk of BP based on the high similarity between Guillain–Barré syndrome and BP. There is currently a lack of evidence for HPV protein sequences shared with human proteins which are associated with the peripheral nervous system. Myelin basic protein, an important antigen for T cells and CNS demyelinating disease, was reported to have a region with a structural similarity to human papillomavirus peptides (35). Furthermore, inflammatory cerebrospinal fluid findings are present in BP, suggesting that it is a generalized central nervous affliction rather than a disease of the peripheral nervous system (36).

Several limitations should be considered when interpreting our findings. First, the epidemiological evaluation of a true HPV infection is challenging because many cases are not clinically recognized. Using retrospective ICD-9-based methods to select study groups of HPV infection may have a selection bias because we can only identify symptomatic patients who required medical attention. A large proportion of HPV infections may be asymptomatic; therefore, individuals who were “clinically unrecognized” could be ignored and may have been included in the comparison group. However, if HPV is associated causally with subsequent BP, misclassification would bias the estimated adjusted HRs toward the null. Therefore, for a better ascertainment of HPV exposure, we excluded patients who were diagnosed with HPV without associated treatment procedures for warts. The purpose of accruing only those patients who received associated treatment into the HPV group was to validate the diagnosis of the physician and to avoid a misclassification bias. However, some patients with an HPV infection with low-grade cervical lesions not requiring treatment might have been enrolled into the control group; similarly, such misclassification may bias our results to null, and thus our findings are acceptable. Second, the ICD-9 codes range from cutaneous benign infections to cervical and anal lesions. Grouping conditions included mucosotropic (Alpha-papillomavirus) non-carcinogenic HPVs (genital warts) to those caused by carcinogenic HPVs as well as to those in a different genus (Beta- or Gamma-papillomavirus) (plantar warts), and this is an inherent limitation of NHIRD because the ICD codes are broad but cannot provide detailed information for biological subgroup analysis. Similarly, methods using ICD-9 codes to identify cases lack information on HPV infection disease severity, which might also lead to an estimated bias because the baseline severity also affects the outcome. However, the methods applied in this study could be a proxy for HPV infection. This is because patients who request medical aid are symptomatic related to pathogenic HPV infections (either cutaneous type or mucosal type or both). These pathogenic effects of HPV infections should be prevented or treated. To overcome these limitations, further studies linking NHIRD to other disease-specified databases are needed. Third, this was a mono-country evaluation, and therefore our findings may not be applicable to non-Asian ethnic groups. Considering possible ethnic and geographical differences in the incidence and serotypes of HPV, further studies should be conducted in other ethnic groups. Fourth, HPV vaccination may also alter the immune system of the host. There are no data regarding HPV vaccination in NHIRD because HPV vaccination is a self-paid service, not covered by the NHI, and is therefore not included in the NHIRD.

In conclusion, this population-based cohort study demonstrated a higher risk of BP in patients with a previous symptomatic HPV infection, and the association was consistent in three different models. The risk was more prominent in the elderly, those with hypertension, or those with COPD. Further studies are required to clarify the underlying biological mechanisms of these associations. HPV vaccination could be another important issue for preventing BP in patients with relevant demographic characteristics and co-morbidities.

## Data Availability Statement

The data analyzed in this study is subject to the following licenses/restrictions: the data source used for this study was the claims data from Taiwan's National Health Insurance Research Database (NHIRD). Analysts had full access to all data in the study and takes responsibility for the integrity of the data and the accuracy of the data analysis. Data are available from the National Health Insurance Research Database (NHIRD) published by Taiwan National Health Insurance (NHI) Bureau. Due to legal restrictions imposed by the government of Taiwan in relation to the “Personal Information Protection Act,” data cannot be made publicly available. Requests for data can be sent as a formal proposal to the NHIRD (http://nhird.nhri.org.tw). Requests to access these datasets should be directed to http://nhird.nhri.org.tw.

## Ethics Statement

The studies involving human participants were reviewed and approved by The Institutional Review Board of the China Medical University in Taiwan approved this study [CMUH-104-REC2-115-(AR4)]. Written informed consent for participation was not required for this study in accordance with the national legislation and the institutional requirements.

## Author Contributions

Acquisition of data and statistical analysis were conducted by H-TY. Study conception and design were carried out by K-YL, M-CC, JW, Y-MH, RC, and H-TY. Analysis and interpretation of data were performed by K-YL, M-CC, Y-MH, RC, JW, and H-TY. K-YL, M-CC, and Y-MH contributed to writing (original draft preparation). Y-MH, M-CC, and JW contributed to writing (review and editing). All the authors were involved in drafting the article or revising it and approved the final version to be published.

## Conflict of Interest

The authors declare that the research was conducted in the absence of any commercial or financial relationships that could be construed as a potential conflict of interest.

## Publisher's Note

All claims expressed in this article are solely those of the authors and do not necessarily represent those of their affiliated organizations, or those of the publisher, the editors and the reviewers. Any product that may be evaluated in this article, or claim that may be made by its manufacturer, is not guaranteed or endorsed by the publisher.

## Supplementary Material

Supplementary Figure 1 Overview of the study.

Supplementary Figure 2 The receiver operating characteristic curves of the main regression model.

Supplementary Table 1 The baseline characteristics in sensitivity analysis 1.

Supplementary Table 2 The risk of Bell's palsy in sensitivity analysis 1.

Supplementary Table 3 The baseline characteristics in sensitivity analysis 2.

Supplementary Table 4 The risk of Bell's palsy in sensitivity analysis 2.
