# Supplementary figures and images for: The Potential Role of Human Papillomavirus Infection in Bell's Palsy: A Hypothesis-Generating Study Based on a Nationwide Cohort
Source: Front Med (Lausanne). 2021 Sep 1;8:616873. doi: 10.3389/fmed.2021.616873 (PMC8447863; doi:10.3389/fmed.2021.616873)

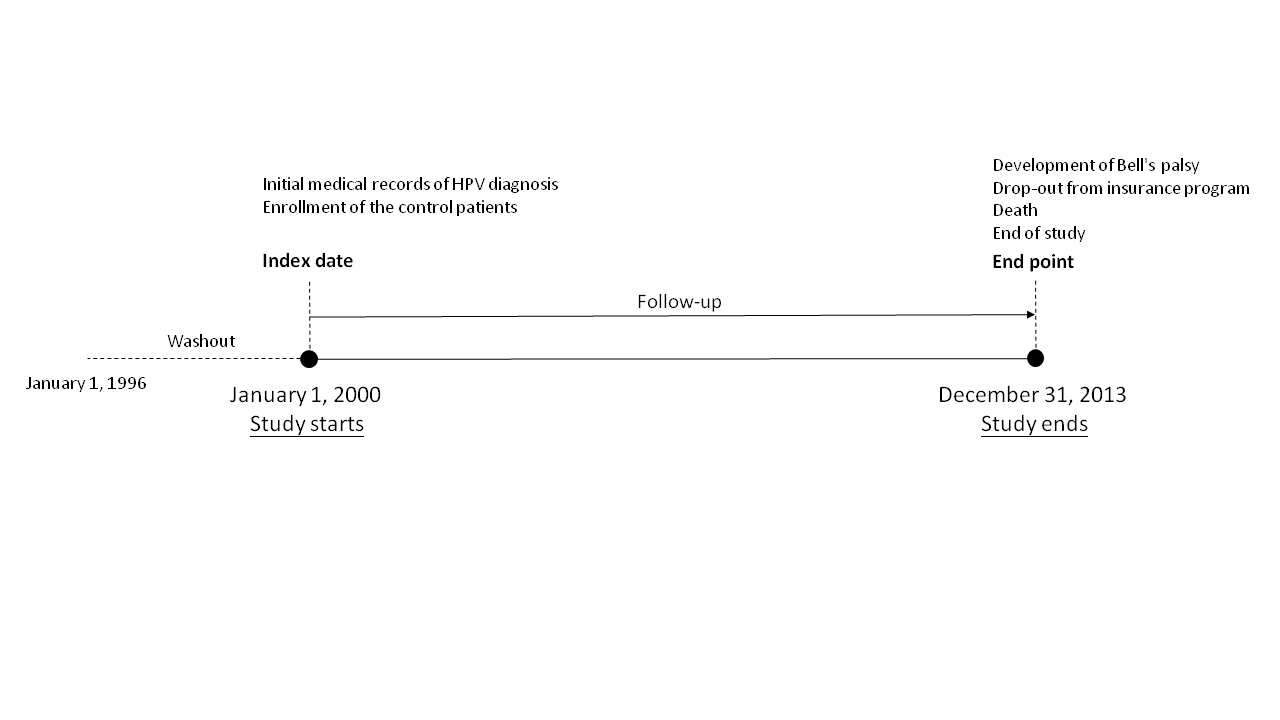

Supplement: Supplementary Figure 1 — Overview of the study. [file Image_1.TIF]

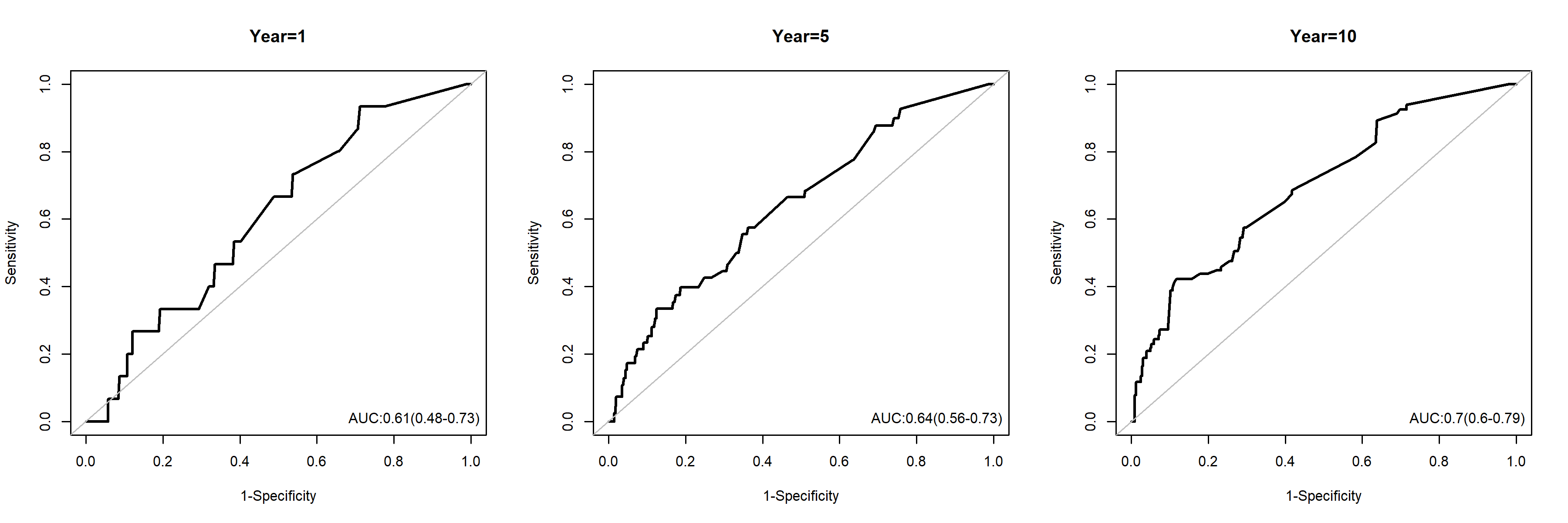

Supplement: Supplementary Figure 2 — The receiver operating characteristic curves of the main regression model. [file Image_2.TIF]
