# Supplementary material for: The Potential Role of Human Papillomavirus Infection in Bell's Palsy: A Hypothesis-Generating Study Based on a Nationwide Cohort
Source: Front Med (Lausanne). 2021 Sep 1;8:616873. doi: 10.3389/fmed.2021.616873 (PMC8447863; doi:10.3389/fmed.2021.616873)
Supplement: Supplementary Table 1 — The baseline characteristics in sensitivity analysis 1. [file Data_Sheet_1.pdf]

Supplementary Table I. The baseline characteristics in sensitivity analysis 1.

	non-HPV N=89 040		HPV N=22 260		
Variables	n	%	n	%	SMD
Sex					<0.001
Female	46648	52%	11662	52%	
Male	42392	48%	10598	48%	
Age, year					
<30	30760	35%	7690	35%	<0.001
30-39	18076	20%	4519	20%	<0.001
40-49	16492	19%	4123	19%	<0.001
50-59	11488	13%	2872	13%	<0.001
60-69	6020	7%	1505	7%	<0.001
70-79	4444	5%	1111	5%	<0.001
≥80	1760	2%	440	2%	<0.001
mean, (SD)	40.4	(16.6)	40.4	(16.6)	0.00
Comorbidities					
hypertension	14804	17%	4029	18%	0.04
diabetes	7380	8%	1991	9%	0.02
hyperlipidemia	12856	14%	4048	18%	0.10
CKD	934	1.0%	325	1.5%	0.04
COPD	7317	8.2%	2291	10%	0.07
alcohol-related illness	1825	2.0%	484	2.2%	0.01
HSV1	4706	5.3%	1961	8.8%	0.14
herpes zoster	2469	2.8%	905	4.1%	0.07
SLE	241	0.27%	97	0.44%	0.03
rheumatoid arthritis	1456	1.6%	455	2.0%	0.03
sicca syndrome	666	0.7%	302	1.4%	0.06
multiple sclerosis	27	0.03%	3	0.01%	0.01
stroke	4463	5%	1127	5%	0.002
Medication					
NSAID	77056	87%	19123	86%	0.02
Steroid	684	0.8%	146	0.7%	0.01

CKD: chronic kidney disease; COPD: chronic obstruction pulmonary disease; HSV1: genital herpes simplex virus; SLE: systemic lupus erythematosus;

SMD: standard mean difference

Supplementary table II. The risk of Bell's palsy in sensitivity analysis 1.

	Bell's palsy						
Variables	n	PY	IR	cHR	(95% CI)	aHR[†]	(95%CI)
HPV							
No	429	488742	0.88	1.00	-	1.00	-
Yes	140	123173	1.14	1.29	(1.07,1.57)**	1.25	(1.03,1.51)*
Sex							
Female	301	322858	0.93	1.00	-		
Male	268	289057	0.93	0.99	(0.84,1.17)		
Age, year							
<30	145	222648	0.65	1.00	-	1.00	-
30-39	116	126598	0.92	1.41	(1.10,1.80)**	1.35	(1.06,1.73)*
40-49	119	113512	1.05	1.61	(1.27,2.06)***	1.41	(1.10,1.80)**
50-59	88	74640	1.18	1.82	(1.40,2.38)***	1.31	(0.98,1.75)
60-69	49	38263	1.28	1.98	(1.43,2.74)***	1.19	(0.82,1.73)
70-79	41	27981	1.47	2.27	(1.60,3.20)***	1.22	(0.81,1.84)
≥80	11	8273	1.33	2.09	(1.13,3.86)*	1.10	(0.57,2.12)
Comorbidities							
hypertension							
No	417	519255	0.80	1.00	-	1.00	-
Yes	152	92660	1.64	2.06	(1.71,2.48)***	1.54	(1.2,1.98)***
diabetes							
No	489	566474	0.86	1.00	-	1.00	-
Yes	80	45441	1.76	2.05	(1.62,2.60)***	1.31	(0.99,1.73)
hyperlipidemia							
No	436	527717	0.83	1.00	-	1.00	-
Yes	133	84198	1.58	1.92	(1.58,2.34)***	1.26	(0.98,1.61)
CKD							
No	563	606820	0.93	1.00	-	1.00	-
Yes	6	5095	1.18	1.28	(0.57,2.87)	0.71	(0.31,1.6)
COPD							
No	498	563832	0.88	1.00	-	1.00	-
Yes	71	48083	1.48	1.68	(1.31,2.15)***	1.21	(0.92,1.59)
alcohol-related illness							
No	555	601547	0.92	1.00	-	1.00	-
Yes	14	10368	1.35	1.48	(0.87,2.52)	1.15	(0.67,1.96)
HSV1							
No	547	580042	0.94	1.00	-		
Yes	22	31873	0.69	0.74	(0.48,1.13)		
herpes zoster							
No	543	596548	0.91	1.00	-	1.00	-
Yes	26	15367	1.69	1.88	(1.27,2.78)**	1.46	(0.98,2.18)

SLE							
No	567	610183	0.93	1.00	-		
Yes	2	1732	1.15	1.25	(0.31,5.01)		
rheumatoid arthritis							
No	558	602502	0.93	1.00	-		
Yes	11	9413	1.17	1.27	(0.70,2.31)		
sicca syndrome							
No	564	607605	0.93	1.00	-		
Yes	5	4310	1.16	1.27	(0.52,3.05)		
multiple sclerosis							
No	569	611785	0.93	1.00	-		
Yes	0	130	0.00				
stroke							
No	540	581312	0.93	1.00	-	1.00	-
Yes	29	30603	0.95	1.02	(0.70,1.48)	1.00	(0.66,1.52)
Medication							
NSAID							
No	95	83224	1.14	1.00	-	1.00	-
Yes	474	528691	0.90	0.79	(0.63,0.98)*	0.78	(0.63,0.98)*
Steroids							
No	563	607356	0.93	1.00	-	1.00	-
Yes	6	4559	1.32	1.42	(0.63,3.17)	1.48	(0.6,3.63)

*: p-value<0.05; **: p-value<0.01; ***: p-value<0.001;

PY: person-years; IR: incidence rate (per 1000 person-years): cHR: crude hazard ratio; aHR: adjusted hazard ratio;

CKD: chronic kidney disease; COPD: chronic obstruction pulmonary disease; HSV1: genital herpes simplex virus; SLE: systemic lupus erythematosus;

ǂ: adjusted by age, hypertension, diabetes, hyperlipidemia, CKD, COPD , herpes zoster, stroke, NSAID and steroids.

Supplementary table III. The baseline characteristics in sensitivity analysis 2.

Variables	non-HPV N=72 132		HPV N=18 033		SMD
	n	%	n	%	
Sex					<0.001
Female	30156	42%	7359	41%	
Male	41976	58%	10494	58%	
Age, year					
<30	24748	34%	6187	34%	<0.001
30-39	11184	16%	2796	16%	<0.001
40-49	13044	18%	3261	18%	<0.001
50-59	11148	15%	2787	15%	<0.001
60-69	5912	8%	1478	8%	<0.001
70-79	4348	6%	1087	6%	<0.001
>=80	1748	2%	437	2%	<0.001
mean, (SD)	41.9	(17.6)	41.9	(17.6)	0.003
Comorbidities					
hypertension	13963	19%	3851	21%	0.05
diabetes	6984	10%	1846	10%	0.02
hyperlipidemia	11969	17%	3768	21%	0.11
CKD	911	1.3%	306	1.7%	0.04
asthma	5522	7.7%	1718	9.5%	0.07
COPD	6590	9.1%	2074	11.5%	0.08
HBV	2449	3.4%	922	5.1%	0.09
HCV	810	1.1%	233	1.3%	0.02
IBD	1517	2.1%	464	2.6%	0.03
alcohol-related illness	1720	2.4%	436	2.4%	0.002
HIV	9	0.0%	6	0.0%	0.01
herpes zoster	2230	3.1%	814	4.5%	0.07
HSV1	3376	4.7%	1506	8.4%	0.15
HSV2	166	0.2%	104	0.6%	0.06
autoimmune disease	1448	2.0%	542	3.0%	0.06

CKD: chronic kidney disease; COPD: chronic obstruction pulmonary disease; HBV: Hepatitis B virus; HCV: Hepatitis C virus; IBD: inflammatory bowel disease; HIV: human immunodeficiency virus; HZ: Herpes zoster infection; HSV1: genital herpes simplex virus; HSV2: non-genital herpes simplex virus; SMD: standard mean difference

Supplementary table IV. The risk of Bell's palsy in sensitivity analysis 2.

Variables	Bell's palsy n	PY	IR	cHR	(95% CI)	aHR	(95%CI)
HPV							
No	379	392312	0.97	1.00	-	1.00	-
Yes	126	99099	1.27	1.32	(1.08,1.61)**	1.27	(1.03,1.55)*
Sex							
Female	235	205410	1.14	1.00	-	1.00	-
Male	270	286002	0.94	0.83	(0.69,0.98)*	0.90	(0.75,1.08)
Age, year							
<30	100	172649	0.58	1.00	-	1.00	-
30-39	67	79095	0.85	1.46	(1.07,1.99)*	1.39	(1.02,1.90)*
40-49	98	94135	1.04	1.79	(1.36,2.37)***	1.51	(1.13,2.01)**
50-59	114	72786	1.57	2.71	(2.07,3.55)***	1.88	(1.4,2.54)***
60-69	61	37375	1.63	2.83	(2.06,3.89)***	1.65	(1.14,2.38)**
70-79	50	27249	1.83	3.19	(2.27,4.47)***	1.71	(1.15,2.55)**
>=80	15	8124	1.85	3.27	(1.90,5.62)***	1.71	(0.95,3.09)
Comorbidities							
hypertension							
No	321	403233	0.80	1.00	-	1.00	-
Yes	184	88179	2.09	2.64	(2.2,3.16)***	1.70	(1.33,2.16)***
diabetes							
No	411	449077	0.92	1.00	-	1.00	-
Yes	94	42335	2.22	2.44	(1.95,3.06)***	1.32	(1.02,1.72)*
hyperlipidemia							
No	354	412399	0.86	1.00	-	1.00	-
Yes	151	79013	1.91	2.24	(1.85,2.71)***	1.21	(0.95,1.53)
CKD							
No	496	486325	1.02	1.00	-	1.00	-
Yes	9	5087	1.77	1.76	(0.91,3.39)	0.89	(0.46,1.75)
asthma							
No	450	456740	0.99	1.00	-	1.00	-
Yes	55	34672	1.59	1.62	(1.22,2.14)***	1.17	(0.87,1.58)
COPD							
No	434	448328	0.97	1.00	-	1.00	-
Yes	71	43084	1.65	1.71	(1.33,2.2)***	0.99	(0.74,1.31)
HBV							
No	486	474756	1.02	1.00	-		
Yes	19	16655	1.14	1.12	(0.71,1.77)		
HCV							
No	498	486637	1.02	1.00	-		
Yes	7	4775	1.47	1.44	(0.68,3.04)		

IBD							
No	490	481689	1.02	1.00	-	1.00	-
Yes	15	9723	1.54	1.52	(0.91,2.55)	1.29	(0.77,2.16)
alcohol-related illness							
No	486	481622	1.01	1.00	-	1.00	-
Yes	19	9790	1.94	1.94	(1.23,3.07)**	1.54	(0.96,2.45)
HIV							
No	505	491330	1.03	1.00	-		
Yes	0	81	0.00				
herpes zoster							
No	484	477594	1.01	1.00	-	1.00	-
Yes	21	13817	1.52	1.51	(0.98,2.34)	1.06	(0.68,1.65)
HSV1							
No	483	468454	1.03	1.00	-		
Yes	22	22958	0.96	0.94	(0.61,1.44)		
HSV2							
No	504	490133	1.03	1.00	-		
Yes	1	1279	0.78	0.77	(0.11,5.44)		
autoimmune disease							
No	492	481725	1.02	1.00	-		
Yes	13	9686	1.34	1.32	(0.76,2.29)		

*: p-value<0.05; **: p-value<0.01; ***: p-value<0.001;

PY: person-years; IR: incidence rate (per 1000 person-years): cHR: crude hazard ratio; aHR: adjusted hazard ratio;

CKD: chronic kidney disease; COPD: chronic obstruction pulmonary disease; HBV: Hepatitis B virus; HCV: Hepatitis C virus; IBD: inflammatory bowel disease; HIV: human immunodeficiency virus; HZ: Herpes zoster infection; HSV1: genital herpes simplex virus; HSV2: non-genital herpes simplex virus;

ł: adjusted by sex, age, hypertension, diabetes, hyperlipidemia, CKD, asthma, COPD, IBD and herpes zoster.
